# The factors of job crafting in emergency nurses: regression models versus qualitative comparative analysis

**DOI:** 10.1186/s12912-024-02035-3

**Published:** 2024-06-03

**Authors:** Yu Wang, Qiaofang Yang, Luwen Wang, Qingwei Zhang, Yingli Li

**Affiliations:** 1 College of Medicine, Jiaxing University, Jiaxing, China; 2 Department of Nursing, Fuwai Central China Cardiovascular Hospital, Zhengzhou, China; 3 Heart Failure Ward, Fuwai Central China Cardiovascular Hospital, Zhengzhou, China

**Keywords:** Job crafting, Emergency nurses, Hierarchical regression model, fsQCA model, Factors, Configuration

## Abstract

**Background:**

Job crafting is defined as a series of proactive behaviors exhibited by employees in order to balance work resources and needs, which has a significant positive impact on the nurses. It is necessary to find the core factors that influence the job crafting, as emergency nurses deal with the most complex tasks, so as to improve their job satisfaction.

**Objectives:**

To investigate the core factors of job crafting among emergency nurses.

**Methods:**

A cross-sectional design was used in the study. A total of 255 nurses were recruited from two hospitals in Zhengzhou and Shenzhen, China in December 2021. 255 nurses completed an online questionnaire. Hierarchical regression models and fsQCA models were used to explore the factors influencing job crafting among emergency nurses and helped us to identify core factors.

**Results:**

The hierarchical regression model and the fsQCA model found that the occupational benefit, psychological empowerment, and research experience were the core factors affecting their job crafting. Job involvement was not significant in the regression model, but the QCA model indicated that it needs to be combined with other factors to impact on job crafting. The QCA model uncovered seven key conditional configurations that led to high and low job crafting among emergency nurses, explaining 80.0% of the results for high job crafting and 82.6% of the results for the low job crafting, respectively.

**Conclusions:**

The results of this study provide valuable insights into the job crafting experienced by emergency nurses. Junior emergency nurses should be granted a high level of psychological empowerment without assigning them overly complex tasks, such as research tasks, as these challenges can stop their job crafting. Intermediate and senior emergency nurses, on the other hand, can be assigned research tasks coupled with high psychological empowerment to enhance their job crafting.

## Introduction

As diseases become more complex and technology evolves rapidly, nursing work is changing, which has posed new tasks and demands for the nurses these days. It was noted that employees should work stepwise in the traditional human resource management system. However, in recent years, researchers have encouraged employees to change their jobs in line with the reality of their work. ‘Job crafting’ is an employee-focused approach to job redesigning [1].Job crafting is defined as a series of proactive behaviors exhibited by employees in order to balance work resources and needs, with the aim of aligning their work with their own preferences, motivations, and passions [2].YEPES-BALDÓ surveyed 530 Spanish nurses and found that the job crafting score was 3.17 ± 0.66 [3]. BAGHDADI conducted a survey among 594 nurses in Saudi Arabia, reporting the job crafting score was 3.54 ± 0.50 [4]. In summary, it is observed that there are variations in the level of job crafting among nurses, which is generally at a moderate level.

Wrzesniewski first proposed job crafting, suggesting that employees could engage in job crafting in three dimensions: cognitive, task, and relational [1]. Job demand-resource was further integrated by Tims and Bakker, and it suggested that all working conditions can be classified as job demands or resources [5, 6], which includes four dimensions: reducing hindering job demands, increasing challenging job demands, increasing structural job resources and increasing social job resources. Promoting-focused job crafting has been shown to be positively related to job involvement and satisfaction, and negatively related to burnout, depression, and illness [7, 8]. Furthermore, studies conducted in the field of career development have shown that promotion-focused job crafting is positively related to career competency and career promotion [9]. Emergency nurses encounter more complex clinical environments and problems than other departments. The emergency department serves as the first line of defense in hospitals to save patients’ lives. However, it is also the department where adverse events such as nurse-patient disputes and complaints are most likely to occur. According to reports, emergency nurses experience higher occupational stress than other departments, which can lead to dissatisfaction with nursing work among nurses, and even turnout [10]. This, in turn, will result in a shortage of emergency nurses, further increasing the occupational stress on those remaining in the emergency department. Furthermore, nurses face additional research tasks and pressures in addition to solving clinical problems in China. So, we defined emergency nurse’s hindering and challenging demands as a research experience, professional position, number of night shifts, etc.

Autonomy is an important work feature, as it can be associated with a better capacity to handle stress [6]. Structural and psychological empowerment are two categories of authority that can give nurses some autonomy at work. Psychological empowerment as an extension of structural empowerment, focuses on the internal feelings of an individual toward organizational empowerment. Spritzer defined psychological empowerment as a sense of control over one’s work environment, which includes the dimensions of impact, meaning, competence, and self-determination [11]. Increased psychological empowerment of nurse has been shown to lead to job satisfaction and more positive organizational behavior [12]. The sense of control over their work behaviors can be increased if nurses have a stronger psychological empowerment. They feel more confident acquiring the required knowledge and skills to do their job, and also have a greater perception of their impact on organizational management, decision-making, and performance [13]. This helps nurses take the initiative toward rebuilding the organizational relationships and actively redesigning work content to address the challenges on the job [14]. Therefore, we defined the work resources as the level of psychological empowerment given to nurses by their organizations.

Occupational benefits are internal, personal motivators that nurses blend with their good work experiences and cognitive assessments [15]. It can also result in greater job satisfaction, the promotion of creative behavior, and a reduction in burnout and turnover intentions [16]. Occupational benefits reflect how satisfied employees are with the organization’s management, which has a significant impact on whether or not they want to stay. According to the JD-R theory, nurses’ job crafting has resulted in favorable occupational benefits for them. Emergency nurses are often faced with critically ill patients, and their nursing work demands a high level of expertise. Correspondingly, the rehabilitation of patients can bring them a sense of accomplishment and self-worth, providing a strong sense of occupational benefits [17]. In order to continually obtain this feeling, emergency nurses may engage in job crafting to reshape their work.Their practice reflects how they view the rewards and advantages of their profession, which supports their involvement in their work.

Job involvement, as a type of work commitment, is the psychological recognition of an individual’s work. It is more linked with the satisfaction of internal needs.From the perspective of an organization, Pfeffe stated that job involvement is crucial for motivating employees [18]. However, there is a disagreement at the moment on whether or not job involvement affects individual performance [19]. On the other hand, some studies have shown that job involvement is the only variable associated with the nurses’ intention to continue working in their current position, not withstanding their work environment [20]. When demands exceed resources, employees feel immense pressure, leading to negative emotions and affecting job involvement [21]. Thus, this suggests that work involvement may arise from job crafting. From this, we hypothesized that emergency nurses with high levels of job involvement may pay more attention to the details of work, which in turn identifies work processes or tasks that need to be improved and triggers job crafting. Therefore, it is worth to be further analyzed whether job involvement would have an impact on job crafting.

Due to the growing complexity of the nurses’ work, one antecedent variable cannot fully explain the causes of the findings. Regression model could only examine the net effect of one of the independent variables on outcomes rather than exploring complex causal relationships between the antecedent variables [22]. The configuration theory is based on the idea of sets, as it allows the analysis of configuration effects generated by multiple conditional variables of the organization’s management [23]. This study used both regression modeling and fsQCA modeling. fsQCA adds depth by showing complex paths to job crafting, where variables can combine differently to explain outcomes. Even non-significant regression variables can impact job crafting in fsQCA. Combining both methods gives a comprehensive view of why emergency nurses reshape their work, revealing intrinsic motivations. Therefore, the aim of our study is to explore the core factors influencing job crafting in emergency nurses through a hierarchical regression model and fuzzy set qualitative comparative analysis.

## Methods

### Sample

A convenience sampling method was applied in the study, an online questionnaire was sent to the emergency nurses through a mobile program in the cities of Shenzhen and Zhengzhou, China, in March 2023. The inclusion criteria were: working in the emergency department; working for more than 1 year; voluntary participation, and informed consent. The exclusion criteria were: trainee nurses, vacation nurses. Based on sample size estimation principles, this study calculated the required sample size to be 5–10 times the number of independent variables. We calculated a required sample of 110–220, given 22 independent variables. Considering a 20% invalid questionnaire rate, we distributed 280 questionnaires.

### Instruments

#### General information questionnaire

The general information questionnaire was designed by the researcher and includes gender, age, monthly income, etc.

#### Perceived occupational benefits Questionnaire[24]

The questionnaire contains 5 dimensions and 29 items. The questionnaire had a 5-point Likert scale, with 1 indicating “strongly disagree” and 5 indicating “strongly agree”. No reverse scoring items were available for the scale. The scores represented the nurses’ perception of professional benefits along with a Cronbach’s alpha of 0.96 in the study.

#### Job crafting Scale[2]

With 21 items, the scale is scored on a 5-point Likert scale. Here, higher scores indicate better job crafting. The scale comprises four dimensions: increasing structural work resources, increasing social work resources, increasing challenging work demands, and decreasing hindering work demands. The Cronbach’s alpha coefficient is 0.93 in the study.

#### Psychological empowerment Scale[25]

This scale includes 4 dimensions: work meaning, autonomy, self-efficacy, and work influence. Each dimension has 3 items under it, totaling 12 items. The 5-point Likert scale is used, where a higher total score indicates a higher degree of PE. The Cronbach’s alpha coefficient of this scale was tested to be 0.94.

#### Job involvement Scale[26]

The scale consists of 10 items on a 5-point Likert scale, with items 2 and 7 being reverse-scored. The level of score indicates the extent of job involvement. The Cronbach’s alpha coefficient of the scale in the study was tested to be 0.82.

### Date collection

Researchers contacted nursing management personnel from various hospitals to obtain their consent and support. Nurses were informed about the research objectives, significance, and principles of anonymity and then shared the questionnaire links to those who agreed to participate. The survey was set up through a survey platform, and to ensure quality, all questions were mandatory. Each nurse gets the link via WeChat and is limited to one response. After the survey concluded, a total of 280 questionnaires were collected. Among them, those questionnaires with identical responses for all items and completion times less than 3 min were excluded. Ultimately, 255 questionnaires were valid, resulting in an effective response rate of 91.07%.

### Statistics

SPSS 24.0 software was used to analyze the data; it was described using frequencies, percentages, or means ± standard deviations, depending on the data type. Comparisons between groups were analyzed using independent samples t-test or one-way ANOVA with a test level of α = 0.05. The paper looks at the amount of variance explained by demographic factors on Job crafting(R^2^) in the one-way analysis of the first level of the hierarchical regression model. In the second level of the hierarchical regression model, other factors that may affect job crafting are integrated to observe the amount of variance explained variance (ΔR^2^).

Qualitative comparative analysis (QCA ) is a new method for analysing complex causal relationships in histological problems based on Boolean algebra and set theory.QCA is a new method that combines quantitative and qualitative analysis, providing strong support for studying the configuration problem. Based on variable type, QCA is divided into csQCA, mvQCA, and fsQCA. fsQCA has been selected in this paper to analyze the configuration effects of job crafting factors among emergency nurses. QCA requires the calibration of the original data into the set. Calibration is the process of assigning sets to cases. Sociodemographic variables that were meaningful for univariate analysis were included in the analysis of the fsQCA model. In this case (Research experience: 0 = None; 1 = have), (Professional Position: 0 = Advanced; 0.5 = Intermediate; 1 = Junior). The fuzzy set requires setting three critical values according to the theoretical or conceptual settings: fully affiliated, crossover, and fully unaffiliated, and with the affiliation of the transformed set between 0 and 1. For the continuous variables, the 0.05th, 0.5th and 0.95th percentile of the data were taken and substituted into the Calibrate function (x, fully in, crossover, fully out).It was analyzed whether the antecedent variable was necessary for job crafting in emergency nurses before the sufficient analysis. If the antecedent variable was greater than 0.9 [27], it was considered necessary. A truth table for sufficient analysis was constructed using fsQCA 3.0, with consistency set to 0.8 and frequency set to 1. If variables appear in both parsimonious and intermediate solutions, they are called core conditions. If they only appear in intermediate solutions, they are called edge conditions [28].

## Results

### Descriptive and univariate analysis

A total of 255 nurses were included, of which 228 (89.4%) were female and up to 209 (82.0%) had no research experience. The results demonstrated that research experience and job position were considered influential variables for job crafting among emergency nurses. Also, the differences were all statistically significant (*p* < 0.05), as seen in Table 1.

**Table 1:** Comparison of Job Crafting scores of emergency nurses by demographic characteristics(*N* = 255)

Variable		*N*(%)	F/t	*P*	Variable		*N*(%)	F/t	*P*
Gender			0.674^a^	0.505	Month Income			2.469	0.063
	Male	27(10.6)				2000-4000RMB	17(6.7)		
	Female	228(89.4)				4001-6000RMB	33(12.9)		
Age			0.674	0.569		6001-8000RMB	39(15.3)		
	20–30	118(46.3)				8000RMB and above	166(65.1)		
	31–40	110(43.1)			Number of nightshifts			1.494	0.217
	41–50	25(9.8)				<1	28(11)		
	51	2(0.8)				1–5	93(36.5)		
Education level			1.678	0.189		6–10	93(36.5)		
	College and below	39(15.3)				11 and above	41(16.1)		
	Bachelor’s degree	209(82)			Professional Position			4.412	0.013
	Master degree and above	7(2.7)				Junior	129(50.6)		
Employment Type			1.16	0.326		Intermediate	116(45.5)		
	Labor Dispatch	5(2)				Advanced	10(3.9)		
	Contract system	180(70.6)			Research experience			4.013 ^a^	< 0.001
	Personnel agent	3(1.2)				Have	46(18)		
	Formal establishment	67(26.3)				None	209(82)		

Note: a = t

### Hierarchical regression models

A variance variation of 0.047 was obtained after entering the sociodemographic factors into the regression model, with significance in the one-way analysis. In the paper, it was shown that nurses with no research experience had a lower job crafting than nurses with research experience(*P*<0.05) In the second step, three psychological variables were added, and it was found that a variance variation of 0.283 was obtained. The significant variables were research experience (β=-0.135, *p* < 0.05), perceived occupational benefits (β = 0.177, *p* < 0.05), and psychological empowerment (β = 0.317, *p* < 0.001), as can be seen in Table 2.

**Table 2:** Hierarchical regression model with the variable job crafting

Model	Variable	Non-standardized		Standardized	t	P	R^2^	ΔR^2^
		β	SE	β				
Model1	constant	90.2	3.242		27.826	< 0.001	0.047**	-
	Research experience							
	None	-4.871	1.891	-0.179	-2.576	0.011		
	Professional Position							
	Junior	-5.513	3.79	-0.263	-1.455	0.147		
	Intermediate	-4.964	3.683	-0.236	-1.348	0.179		
Model2	constant	46.823	5.281		8.867	< 0.001	0.326**	0.283**
	Research experience							
	None	-3.671	1.595	-0.135	-2.302	0.022		
	Professional Position							
	Junior	-3.296	3.225	-0.157	-1.022	0.308		
	Intermediate	-2.815	3.131	-0.134	-0.899	0.369		
	Psychological empowerment	0.437	0.119	0.317	3.668	< 0.001		
	Perceived Occupational Benefits	0.12	0.053	0.177	2.244	0.026		

Note: **P<0.01

### Necessary analysis

The consistency score was considered necessary for evaluating whether the antecedent variable was available as the outcome variable. The consistency score is similar to the significance of the coefficient in a regression model. It represents the extent to which the outcome has to rely on the antecedent variable. In this study, no variable existed as a necessary condition since none of the antecedent variables reached 0.9. Refer to Table 3 for details.

**Table 3:** Necessity analysis for quality of life

Variable	Job Crafting		~Job Crafting	
	Consistency	Coverage	Consistency	Coverage
Psychological empowerment	0.835	0.799	0.592	0.635
~Psychological empowerment	0.619	0.575	0.813	0.846
Job involvement	0.807	0.746	0.608	0.631
~Job involvement	0.601	0.578	0.755	0.814
Perceived Occupational Benefits	0.848	0.772	0.648	0.661
~Perceived Occupational Benefits	0.628	0.614	0.777	0.851
Professional Position	0.859	0.549	0.885	0.634
~Professional Position	0.427	0.768	0.371	0.747
Research experience	0.229	0.599	0.137	0.401
~Research experience	0.771	0.444	0.863	0.556

Note: Condition needed: consistency ≥ 0.90;“~” indicates low

### Sufficient analysis

Six conditional configurations that generated high job crafting and six conditional configurations that generated low job crafting were analyzed together. The twelve configurations were sufficient conditions to constitute high and low job crafting for the emergency nurses. The overall solution coverage of high and low job crafting was 0.800 and 0.826, as seen in Table 4. This shows that the twelve configurations explain 80% of the results for high job crafting and 82.6% for low. The paper remove unique coverage of less than 0.1 for the configuration because it was hard to cover 10% of the samples. Three configurations of high job crafting and four configurations of low job crafting for emergency nurses are obtained.The raw coverage of H1 was 0.106 in the high job crafting configuration, which meant that this configuration could explain 10.6% of the sample.Among the low job crafting configurations, it was found that L2 had the highest raw coverage. The configurations are elaborated as follows: ① H1: high psychological empowerment + high job involvement + junior professional position + no research experience. ② H2: high psychological empowerment + high job involvement + have research experience. ③H3:high job involvement + high perceived occupational benefits + senior professional position + no research experience. ④ L1: low perceived occupational benefits + senior professional position + no research experience. ⑤L2: low psychological empowerment + senior professional position + no research experience. ⑥L3: high job involvement + senior professional position + low perceived occupational benefits. ⑦L4: low job involvement + senior professional position + research experience.The most relevant pathway or combination to explain low job crafting was L2 (raw coverage = 0.038; consistency = 0.879), which explained 64.3% of the cases. The most relevant pathway or combination to explain high job crafting was H1 (raw coverage = 0.106; consistency = 0.798), which explained 60.1% of the cases.Refer to Table 4 for details.

**Table 4:** Summary of the sufficient conditions for the intermediate solution of low & high job crafting

Variable	High Job Crafting			Low Job Crafting			
	H1	H2	H3	L1	L2	L3	L4
Psychological empowerment	**X**	**X**			$\otimes$		
Job involvement	**×**	**X**	**X**			**×**	$\otimes$
Perceived Occupational Benefits			**X**	$\otimes$		$\otimes$	**×**
Professional Position	**×**		**×**	**×**	**×**	**×**	**×**
Research experience	$\otimes$	**X**	$\otimes$	$\otimes$	$\otimes$		
Unique coverage	**0.601**	**0.176**	**0.530**	**0.620**	**0.643**	**0.459**	**0.482**
Raw coverage	**0.106**	**0.080**	**0.035**	**0.014**	**0.038**	**0.001**	**0.023**
Consistency	**0.798**	**0.937**	**0.833**	**0.883**	**0.879**	**0.911**	**0.910**
**Overall solution** **consistency**	**0.846**			**0.828**			
**Overall solution** **coverage**	**0.800**			**0.826**			

Note: “•”indicate the presence of a condition; “$\otimes$”indicate its absence; Large circles indicate core conditions; small ones indicate peripheral conditions. Blank spaces indicate “don’t care.”

## Discussions

This study explored the effects of sociodemographic variables, psychological empowerment, occupational benefits, and job involvement, on job crafting among the emergency nurses. The majority of existing studies have concentrated on linear regression models. This neglects the complement of other methods, like the fsQCA model [29]. The study overlooked the synergy between factors if the researcher focuses only on regression models. In helping the researcher to construct intervention plans, the fsQCA models with different pathways formed by the synergy between factors are particularly important [30]. Regression models indicated that research experience, psychological empowerment and occupational benefits were associated with job crafting, which is consistent with existing research [31]. Based on the results of the fsQCA analysis, no necessary conditions for job crafting were found. In terms of the sufficiency analysis, it was found that H1 has the highest coverage, explaining 10.6% of cases after comparing the raw coverage of the three configurations that stimulate high job crafting among emergency nurses. This suggested that the majority of emergency nurses who exhibit high job crafting are influenced by the conditions present in H1, and psychological empowerment is the core condition. For the four paths of the low job crafting, L2 has the highest raw coverage. This indicated that nurses with junior professional position and no research experience who lack sufficient psychological empowerment are unlikely to engage in job crafting. Once again, psychological empowerment emerges as a core condition in this path, reinforcing the results obtained through regression analysis.

We can discuss the professional position and research experience from “increasing the challenging work demands” and “lessening hindering work demands”, based on the job crafting theory [6]. Since junior emergency nurses were newly exposed to clinical nursing work along with the special characteristics of emergency nursing, they faced higher work stress when addressing clinical problems that were primarily acute and serious. The problems mentioned above can also serve as a cause of stress that constantly reduces self-efficacy in the emergency care process. Additionally, the junior emergency nurses lack knowledge and experience in nursing research when they were students, and the research ability of nurses working in clinical settings is low in China [32]. Most nurses are under pressure to perform the job guided by the goal of job position improvement [33], and the inner drive to explore and solve research problems was insufficient [34]. If the nurses were assigned to research work by the organization, the work demands would inevitably exceed their abilities, leading to work hindrances. Junior nurses experience less pressure about job position improvement, and they may be fearful of research work. This may create defensive job crafting by lessening hindering work demands. Although this helps them to accomplish their clinical goals, it may reduce their work motivation and job involvement. The second dimension of job crafting is about increasing the challenging work demands. It has been revealed that a lack of challenging work may lead to absences and job dissatisfaction. Research tasks, as a challenge, may lead to the creation of job crafting for advanced practice nurses. By expanding task boundaries and increasing challenging work demands, the nurses have contributed to the organization. It was shown that challenge demands were associated with goal achievement and work motivation [6]. The H2, with research experience as a core condition, confirmed that the high job crafting was associated with the individuals’ promotion of “challenge demands”.

The high level of psychological empowerment in nurses was a core condition that influenced the level of job crafting on both core configurations (H1&L2). From regression models, it was also seen that psychological empowerment was a core element affecting job crafting (β = 0.317, *p* < 0.001). This means that the job crafting gradually increased with the psychological empowerment. The self-determination theory suggests that autonomous motivational orientation, as opposed to control motivational orientation, would be more beneficial in addressing the basic psychological needs. A high level of psychological empowerment as a variable of autonomous motivational orientation could enhance individual job performance and job ability [35]. The resource conservation theory states that employees can experience higher psychological security as they sense higher levels of psychological empowerment through a cycle of resource loss and gain spirals. These positive job resources assist them in conserving and building more resources to cope with the prospect of poor career outcomes and job demands [36], which precisely confirm the findings of this paper.

In the emergency departments, nurses face a large number of job challenges on a daily basis. The ability of nurses to cope better with these challenges is closely related to perceived occupational benefits. Emergency nurses who perceive high levels of empowerment were better able to respond to the work challenges [37]. With increased resources for employees to do their jobs, a positive impact was bound to be seen on their psychological empowerment [14]. By acquiring and conserving resources, nurses are most likely to achieve the most appropriate match between people and job demands. Nurses who are motivated and empowered can develop more job crafting [38]. If this goal is achieved, it would increase nurses’ satisfaction and thus would promote job crafting resulting in a virtuous circle. To summarize, the results of both the QCA and the regression model demonstrated that the psychological empowerment was a core condition that influenced job crafting among the emergency nurses. From H1, H2 and L2, it can also be proved that high psychological empowerment was the core condition for a high job crafting, regardless of whether or not they had done clinical research work.

The identification with one’s job based on its potential to meet one’s needs and expectations is called job involvement. The job involvement has been mentioned in the literature as a reason why nurses feel so committed to their jobs [39]. Nurses with a high level of job involvement deliberately consider their work an important part of their lives. Whether or not they can feel good about themselves is closely related to their personal work. Thus, a healthy management structure should consider job involvement as an important predictor of organizational productivity. This attitude of nurses toward their jobs should be promoted [20]. However, the regression model shows that job involvement does not influence job crafting. Meanwhile, the H1-H3 all indicated that job involvement was an impact on job crafting, and two of these paths indicated that job involvement was a core condition for a high job crafting. Job involvement is influenced by the worker’s identification with the job they are doing. This is, in turn, derived from whether the job can meet the needs of the worker or not. This means that once a nurse has job involvement, a balance between job resources and demands can be reached and the nurse’s job crafting would be suspended. The QCA model revealed that job involvement may need to have an impact on job crafting in conjunction with the other factors. Several studies have also shown that job involvement is correlated with psychological empowerment [40]. The impact of nurses’ job involvement on their job crafting needs to be further explored.

The occupational benefits of nurses is a cognitive assessment of their feelings about the content of their work, which comes from their internal traits and the external work environment [41]. Job crafting is a positive behavior for individuals to balance job demands and resources. It can help individuals use available job resources to cope with the stress of job demands and achieve higher levels of job performance [42]. It enables the nurses to perceive occupational benefits. The H3 demonstrated that both the occupational benefits and the job involvement must be maintained at high levels for junior emergency nurses to lead to a high job crafting. The existence of a low occupational benefits and job involvement will inevitably lead to low job crafting, as seen from L3 and L4. When junior emergency nurses perceive occupational benefits, it may lead to more job autonomy which results in increased psychological recognition of their work [43]. High levels of job involvement allow nurses to be fully immersed in their work, improving efficiency and quality. With increased efficiency and quality, nurses can obtain more job performance and achievement, in turn bringing them stronger occupational benefits.

### Limitations

Although the study obtained some results that could be effective in improving the job crafting for the emergency nurses, the limitations of the study are as follows. First, this cross-sectional survey was conducted with emergency nurses in China, which may limit the extension of these results to other regions. And convenient sampling techniques and the non-calculated sample size of the study limits the generalizability. Secondly, the cross-sectional study does not allow for the detection of possible changes in the levels of job crafting in each participant over time. Lastly, the data were collected from participants using self-report measures, and thus may not reflect their true feelings.

### Implications for the profession

The findings of the paper provided two important insights for motivating job crafting in emergency nurses. Firstly, we recognized challenge demands have a significant contribution to job crafting. As such, nursing managers in emergency departments should assign nurses challenging tasks, such as participating in nursing research. These challenges not only stimulate nurses’ potential but also foster their personal growth. However, it’s crucial to align these challenging work demands with commensurate rewards, such as promotion in position, bonus allocation, etc. Meanwhile, it is necessary to give adequate psychological empowerment and cultivate a proper understanding of challenge demands such as research tasks to inspire job crafting in the nurses. This approach will encourage nurses to more actively engage in job crafting, continually improving their work efficiency. Secondly, emergency nursing managers should should carry out a layered method and focus on the main job demands of the nurses at different levels. Junior nurses experience more difficulty in facing the challenges brought by clinical work, which may not deal with the busy and ever-changing work of the emergency department. Thus, special attention should be paid to their psychological endurance and work stress to prevent job burnout and turnover when assigning research or other challenging tasks to them. For senior nurses, management should provide more psychological empowerment, making them feel trusted and respected by the organization. An organization that meets the staff needs and promotes staff development on priority allows nurses to perceive occupational benefits, enhances their sense of emotional belonging, and lastly, boosts the job crafting with a rise in job involvement [44]. Nurses will be more proactive in participating in work planning and implementation, actively adjusting and optimizing work processes to better meet the various challenges in the emergency department.

## Conclusions

The study explored various influencing factors on the job crafting of emergency nurses through hierarchical regression and fsQCA models. Both the models have demonstrated that research experience, psychological empowerment, and occupational benefits were predictors of job crafting, along with high levels of psychological empowerment being the core condition on the higher paths (H1 & S2). Based on research findings, junior emergency nurses should be granted a high level of psychological empowerment without assigning them overly complex tasks, such as research tasks, as these challenges can stop their job crafting. Intermediate and senior emergency nurses, on the other hand, can be assigned research tasks coupled with high psychological empowerment to enhance their job crafting.

## Data Availability

The datasets used and/or analyzed during the current study are available from the corresponding author on reasonable request.

## References

[1] Wrzesniewski A, Dutton JE (2001). Crafting a job: revisioning employees as active crafters of their work. Acad Manage Rev.

[2] Tims M, Bakker AB, Derks D (2012). Development and validation of the job crafting scale. J VOCAT BEHAV.

[3] Yepes-Baldó M, Romeo M, Westerberg K, Nordin M (2018). Job Crafting, Employee Well-being, and Quality of Care. WESTERN J NURS RES.

[4] Baghdadi NA, Farghaly Abd EL, Aliem SM, Alsayed SK (2021). The relationship between nurses’ job crafting behaviours and their work engagement. J NURS MANAGE.

[5] Bakker AB, Demerouti E (2017). Job demands–resources theory: taking stock and looking forward. J OCCUP HEALTH PSYCH.

[6] Tims M, Bakker AB. Job crafting: towards a new model of individual job redesign. SA J IND PSYCHOL 2010, 36(2).

[7] Rudolph CW, Katz IM, Lavigne KN, Zacher H (2017). Job crafting: a meta-analysis of relationships with individual differences, job characteristics, and work outcomes. J VOCAT BEHAV.

[8] Lichtenthaler PW, Fischbach A (2019). A meta-analysis on promotion- and prevention-focused job crafting. EUR J WORK ORGAN PSY.

[9] Akkermans J, Tims M (2017). Crafting your Career: how Career competencies relate to Career Success via Job crafting. Appl Psychol.

[10] Yinghao Z, Dan Z, Qi L, Yu W, Xiaoying W, Ao F, Lin Z (2023). A cross-sectional study of clinical emergency department nurses’ occupational stress, job involvement and team resilience. INT EMERG NURS.

[11] Spreitzer GM (1995). PSYCHOLOGICAL, EMPOWERMENT IN THE WORKPLACE: DIMENSIONS, MEASUREMENT AND VALIDATION. ACAD MANAGE J.

[12] Jafari F, Salari N, Hosseinian-Far A, Abdi A, Ezatizadeh N (2021). Predicting positive organizational behavior based on structural and psychological empowerment among nurses. COST EFFECT RESOUR A.

[13] Hatamian P, Farsani MA. Predicting Job Satisfaction Based on Personality Traits and Psychological Empowerment in Employed Middle-Aged and Elderly People. *Sālmand* 2019, 13(4):418–427.

[14] Matsuo M (2019). Personal growth initiative as a predictor of psychological empowerment: the mediating role of job crafting. HUM RESOUR DEV Q.

[15] Hu Y, Hu J, Li L, Zhao B, Liu X, Li F (2020). Development and preliminary validation of a brief nurses’ perceived professional benefit questionnaire (NPPBQ). BMC MED RES METHODOL.

[16] Zhan T, Li H, Ding X (2020). Can social support enhance sense of coherence and perceived professional benefits among Chinese registered nurses? A mediation model. J NURS MANAGE.

[17] Mollanazari M, Shams Z. The Influence of Professional Identity and Outcome Knowledge on Professional Judgment. 2017.

[18] Paullay IM, Alliger GM, Stone-Romero EF (1994). Construct validation of two instruments designed to measure job involvement and work centrality. J APPL PSYCHOL.

[19] Zhang B, Chen JZ. The Summary of Work Engagement,Job involvement and Job Embeddedness. East China Economic Manage 2009.

[20] Nylen-Eriksen M, Grov EK, Bjornnes AK (2020). Nurses’ job involvement and association with continuing current position-A descriptive comparative study. J CLIN NURS.

[21] Jiang D, Ning L, Liu T, Zhang Y, Liu Q. Job demands-resources, job crafting and work engagement of tobacco retailers. FRONT PUBLIC HEALTH 2022, 10. 10.3389/fpubh.2022.925668 PMC9441671 36072378

[22] Furnari S, Crilly D, Misangyi VF, Greckhamer T, Fiss PC, Aguilera RV (2020). Capturing Causal Complexity: Heuristics for Configurational Theorizing. ACAD MANAGE REV.

[23] Rihoux B, Ragin CC. *Configurational comparative methods: qualitative comparative analysis (QCA) and related techniques*: Configurational comparative methods.

[24] Jing H, Xiaohong L (2013). Establishment of a questionnaire of nurses’ perceived professional benefits: reliability and validity assessment. Nurs J Chin People’s Liberation army.

[25] Chaoping L, Xiaoxuan L, Kan S, Xuefeng C (2006). Psychological empowerment: measurement and its effect on emplpyee work attitude in China. ACTA PSYCHOL SIN.

[26] Kanungo RN (1982). Measurement of job and work involvement. J APPL PSYCHOL.

[27] Ming Z, Du Y (2019). Qualitative comparative analysis(QCA) in management and organization research: position, tactics and directions. Chin J Manage.

[28] Fernandez-Garcia D, Moreno-Latorre E, Gimenez-Espert M, Prado-Gasco V (2021). Satisfaction with the clinical practice among nursing students using regression models and qualitative comparative analysis. NURS EDUC TODAY.

[29] Gimenez-Espert M, Valero-Moreno S, Prado-Gasco VJ (2019). Evaluation of emotional skills in nursing using regression and QCA models: a transversal study. NURS EDUC TODAY.

[30] Mei S, Lv J, Ren H, Guo X, Meng C, Fei J, Yuan T, Yue J, Gao R, Song Q (2022). Lifestyle behaviors and depressive symptoms in Chinese adolescents using regression and fsQCA models. FRONT PUBLIC HEALTH.

[31] Harbridge R, Ivanitskaya L, Spreitzer G, Boscart V (2023). Psychological empowerment and job crafting among registered nurses working in public health: a quantitative study. APPL NURS RES.

[32] Wang M, Xie R, Lu Q, Ou J, Gao L (2019). Determinants of research ability among nurses in a tertiary hospital in China: a cross-sectional survey. J Educational Res Reviews.

[33] Gao L, Lu Q, Hou X, Ou J, Wang M (2022). Effectiveness of a nursing innovation workshop at enhancing nurses’ innovation abilities: a quasi-experimental study. NURS OPEN.

[34] Chun T, Shizhe L, Wenrui L, Xiangqun M, Abdi A (2022). Current situation and influencing factors of scientific research innovation activities of professionals in disease control and prevention institutions in Jiangxi Province. Mod Prev Medicin.

[35] Deci EL, Olafsen AH, Ryan RM (2017). Self-determination theory in Work organizations: the state of a science. ANNU REV ORGAN PSYCH.

[36] Zhou H, Chen J (2021). How does psychological empowerment prevent emotional exhaustion? Psychological safety and Organizational Embeddedness as mediators. FRONT PSYCHOL.

[37] Boudrias JS, Morin AJ, Brodeur MM (2012). Role of psychological empowerment in the reduction of burnout in Canadian healthcare workers. NURS HEALTH SCI.

[38] Miller ML. Relationships Between Job Design, Job Crafting, Idiosyncratic Deals, and Psychological Empowerments. *Dissertations & Theses - Gradworks* 2015.

[39] Abd-El AS, Abou HE (2021). The relationship between transformational Leadership practices of First-Line nurse managers and nurses’ organizational resilience and job involvement: a structural equation Model. WORLDV EVID-BASED NU.

[40] Faulkner J, Laschinger H (2008). The effects of structural and psychological empowerment on perceived respect in acute care nurses. J NURS MANAGE.

[41] Zhou H, Zhu Y, Zhang X, Peng J, Li Q, Wang X, Wang L, Cai X, Lan L (2018). Psychological capital and Perceived Professional benefits: testing the Mediating role of perceived nursing work environment among Chinese nurses. J PSYCHOSOC NURS MEN.

[42] Hui C, Hui Y (2021). Application progress on job demand-resources model in the field of nursing. Chin Evidence-Based Nurs.

[43] Jinxia J, Haiyan S, Zhu X, Meimei T (2020). Perspectives of professional benefits among emergency nurses: a qualitative study. J Nurs Sci.

[44] Clegg C, Spencer C (2007). A circular and dynamic model of the process of job design. J OCCUP ORGAN PSYCH.

